# Imaging approaches to assess the therapeutic response of gastroenteropancreatic neuroendocrine tumors (GEP-NETs): current perspectives and future trends of an exciting field in development

**DOI:** 10.1007/s10555-015-9598-5

**Published:** 2015-10-03

**Authors:** Rocio Garcia-Carbonero, Roberto Garcia-Figueiras, Alberto Carmona-Bayonas, Isabel Sevilla, Alex Teule, Maria Quindos, Enrique Grande, Jaume Capdevila, Javier Aller, Javier Arbizu, Paula Jimenez-Fonseca, on behalf of the Spanish Cooperative Group of Neuroendocrine Tumors (GETNE)

**Affiliations:** Medical Oncology Department, Hospital Universitario Doce de Octubre, Center affiliated to the Red Tematica de Investigacion Cooperativa en Cancer (RTICC), Instituto de Salud Carlos III, Spanish Ministry of Science and Innovation, Av. Cordoba km 5.4, 28041 Madrid, Spain; Radiology Department, Complexo Hospitalario Universitario de Santiago de Compostela, Santiago de Compostela, Spain; Hematology and Medical Oncology Department, Hospital Universitario Morales Meseguer, Murcia, Spain; Medical Oncology Department, Hospital Universitario Virgen de la Victoria y Hospital Regional Universitario, Málaga, Spain; Medical Oncology Department, Instituto Catalán de Oncología (ICO), Center affiliated to the Red Tematica de Investigacion Cooperativa en Cancer (RTICC), Instituto de Salud Carlos III, Spanish Ministry of Science and Innovation, Seville, Spain; Medical Oncology Department, Complejo Hospitalario Universitario, A Coruña, Spain; Medical Oncology Department, Hospital Ramon y Cajal, Madrid, Spain; Medical Oncology Department, Hospital Vall d’Hebron, Center affiliated to the Red Tematica de Investigacion Cooperativa en Cancer (RTICC), Instituto de Salud Carlos III, Spanish Ministry of Science and Innovation, Barcelona, Spain; Endocrinology Department, Hospital Puerta de Hierro, Madrid, Spain; Nuclear Medicine Department, Clinica Universidad de Navarra, Navarra, Spain; Medical Oncology Department, Hospital Universitario Central de Asturias, Oviedo, Spain

**Keywords:** Neuroendocrine tumors, Response assessment, Response criteria, Radiological evaluation, Functional imaging

## Abstract

Gastroenteropancreatic neuroendocrine tumors (GEP-NETs) are a family of neoplasms with a complex spectrum of clinical behavior. Although generally more indolent than carcinomas, once they progress beyond surgical resectability, they are essentially incurable. Systemic treatment options have substantially expanded in recent years for the management of advanced disease. Imaging plays a major role in new drug development, as it is the main tool used to objectively evaluate response to novel agents. However, current standard response criteria have proven suboptimal for the assessment of the antiproliferative effect of many targeted agents, particularly in the context of slow-growing tumors such as well-differentiated NETs. The aims of this article are to discuss the advantages and limitations of conventional radiological techniques and standard response assessment criteria and to review novel imaging modalities in development as well as alternative cancer- and therapy-specific criteria to assess drug efficacy in the field of GEP-NETs.

## Introduction

Neuroendocrine tumors (NETs) of the gastroenteropancreatic tract (GEP) are a family of neoplasms with a complex spectrum of clinical behavior. GEP-NETs arise from disseminated endocrine cells that can store and secrete amines in response to different stimuli. The broad anatomical location and heterogeneous biology of GEP-NETs makes their clinical management particularly challenging [1]. Treatment approaches include surgery, locoregional liver-directed therapy, peptide receptor radionuclide therapy (PRRT), and systemic hormonal, cytotoxic, or targeted therapy. Disease localization and extent, resectability of the primary and metastatic disease, tumor biology and dynamics, presence or absence of tumor somatostatin receptors, local expertise and availability of therapeutic options (*e.g.*, PRRT), clinical symptoms, personal preferences, and the patient’s overall health condition are all key factors to properly design an adequate customized treatment plan for each patient.

Treatment options for GEP-NETs have substantially increased in recent years, particularly in the area of systemic medical therapy for advanced disease [2]. Imaging plays a major role in new drug development, as it is the primary tool used to objectively assess tumor response to novel agents. However, current standard response assessment criteria—Response Evaluation Criteria In Solid Tumors (RECIST) [3, 4]—were originally developed to measure cytotoxic chemotherapy efficacy in solid tumors and are suboptimal to evaluate the antiproliferative effects of many new cytostatic agents, particularly in slow-growing tumors such as well-differentiated GEP-NETs. In fact, several agents (*i.e.*, octreotide, lanreotide, sunitinib, and everolimus) induce few, if any, objective responses according to the conventional criteria, but significantly delay tumor growth thereby improving progression-free survival (PFS) of patients [5–8]. To address the limitations of RECIST, a number of alternative response criteria have been proposed for specific types of cancer and therapeutic agents or strategies. At the same time, the extraordinary advances achieved in the field of functional imaging are providing new means for assessing the antitumor activity of different therapeutic approaches. This article aims to discuss the advantages and limitations of current standard radiological techniques and response assessment criteria and to review new imaging modalities that are being developed as well as alternative cancer- and therapy-specific criteria to evaluate drug efficacy in the field of GEP-NETs.

## Imaging assessment of tumor response

### Conventional imaging modalities

Morphological and functional imaging techniques are crucial for characterizing and managing GEP-NETs in clinical practice. Various different imaging modalities are used for screening at-risk populations, detecting primary lesions, assessing the extent of the disease, and evaluating the patient’s response to treatment [5, 9–11]. The choice of which techniques to use depends on clinical presentation and specific tumor features, including location, somatostatin receptor expression, functionality, and proliferation rate [10]. Currently, GEP-NETs are morphologically assessed by computed tomography (CT) and magnetic resonance imaging (MRI). Additionally, various ultrasound (US) approaches (transabdominal, endoscopic, and intraoperative) can be useful for detecting certain primary tumors (*e.g.*, pancreatic) or liver metastasis (Table 1).

**Table 1:** Main morphological and functional imaging modalities available to evaluate response to treatment in patients with GEP-NETs

Imaging modality	Principle/target	Mechanism/radiotracer	Biological correlation	Advantages	Limitations
US	Tissue perfusion and vascularity: – Blood flow – Peak intensity – Time to peak intensity – Area under the curve	DCE: enhanced representation of the vasculature following the administration of microbubbles		– No ionizing radiation – Real-time imaging – Short acquisition time – Inexpensive – Availability	– Whole-body imaging not possible – Contrast agents are limited to vasculature – Operator dependency – Assessment limited to selected targets
CT	Tissue perfusion and vascularity: – Relative blood volume – Relative blood flow – Mean transit time	DCE: changes in density following the administration of iodinated contrast agent	– Vessel density – Vascular permeability – Perfusion	– High spatial resolution – Short acquisition time – Moderately expensive – Availability	– Radiation burden – Poor soft tissue contrast – Assessment limited to selected targets
MRI	Tissue perfusion and vascularity: – Initial curve under the gadolinium curve – Transfer rate and constants – Leakage space fraction – Fractional plasma volume	DCE: contrast average uptake rate in tissues Influenced by transfer rate, extracellular volumes, plasma volume fraction	– Vessel density – Vascular permeability – Perfusion – Tissue cell fraction – Plasma volume	– No ionizing radiation – Excellent soft tissue contrast	– Expensive – Long acquisition time – Low availability – Assessment limited to selected targets – Good patient cooperation required
Scintigraphy	SSTR2	^111^In-pentetreotide	– SSTR2 density	– Whole-body scan possible – Availability – Sensitivity and specificity for staging superior to conventional imaging	– High to moderate affinity to SSTR2 – Low resolution; planar views – Long acquisition time – Evaluation of organs with high physiological uptake (*e.g.*, liver, gut)
SPECT or SPECT/CT	SSTR2	^111^In-pentetreotide	– SSTR2 density	– Tomographic imaging – Combines functional and structural information (SPECT/CT)	– Lower spatial resolution than PET – Long acquisition time – Suboptimal physical resolution of isotopes used for SPECT
PET/CT	SSTR2	^68^Ga-DOTA-TATE	– SSTR2 density	– Whole-body scan possible – High spatial resolution of PET – Short acquisition time – Very high affinity to SSTR – Rapid extraction and clearance – Combines functional and structural information	– Limited to SSTR2 expression – Tumor dedifferentiation and loss of SSTR expression
	SSTR2, SSTR5	^68^Ga-DOTA-TOC	– SUV with IRS of SSTR2A – SUV with tumor-absorbed doses after PRRT	Idem	– Limited to SSTR2 and SSTR5 expression – Tumor dedifferentiation and loss of SSTR expression
	SSTR2, SSTR3, SSTR5	^68^Ga-DOTA-NOC	– SUV with IRS of SSTR2A and SSTR5	Idem – Superior to other ^68^Ga-radiolabeled peptides	– Tumor dedifferentiation and loss of SSTR expression
	Catecholamine transporter and synthesis	^18^F-DOPA	– Urinary levels of 5-HIAA – No correlation with SSTR	– Whole-body scan possible – Greater sensitivity than SRS – Role in negative SRPET and inconclusive conventional imaging – Greater sensitivity in functioning tumors	– Lower sensitivity than ^68^Ga-labeled PET
	Catecholamine transporter and synthesis	^11^C-5-HTP	– Urinary levels of 5-HIAA – No correlation with SSTR	– Same as ^18^F-DOPA	– Very short half-life of radiotracer – Very low availability
	Glucose transporter	^18^F-FDG	– Ki-67 %	– Whole-body scan possible – Poorly differentiated and/or highly proliferative tumors	– Useless in well-differentiated tumors

*CT* computed tomography, *DCE* dynamic contrast-enhanced, *5-HIAA* 5-hydroxyindoleacetic acid, *IRS* immunoreactive score of Remmele and Stegner, *MRI* magnetic resonance imaging, *PET/CT* positron emission tomography/computed tomography, *SPECT* single photon computed tomography, *SPECT/CT* single photon tomography/computed tomography, *SSTR* somatostatin receptors, *SUV* standard uptake value in PET/CT images, *US* ultrasound

**Fig. 1:**
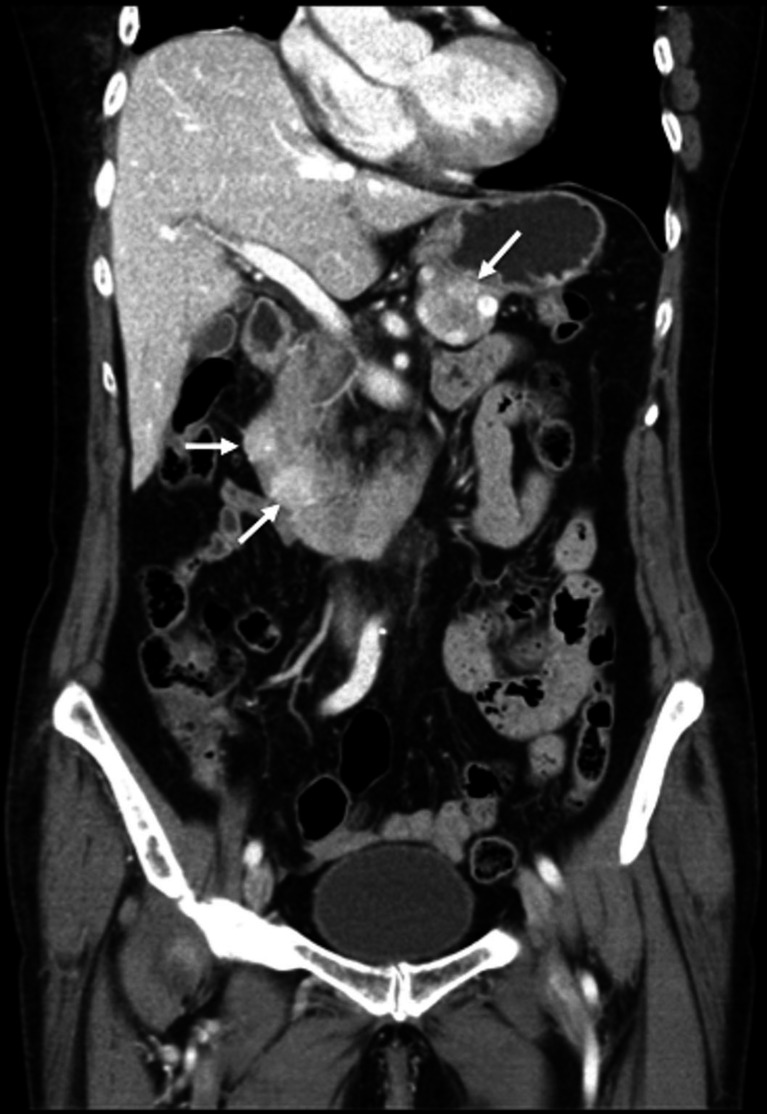
Coronal reformatted arterial phase contrast-enhanced multidetector CT image demonstrates multiple enteropancreatic neuroendocrine tumors (*arrows*) in a patient with MEN-1. These tumors and their metastases are often hypervascular. They are usually more conspicuous in the early arterial acquisition phase

CT is the most commonly used technique for initial tumor localization, staging, therapeutic monitoring, and follow-up of patients with GEP-NETs. Its rapid acquisition process and ability to generate multiplanar reconstructions provide high temporal and spatial resolution (20–200 μm; pixel/voxel dimension <1 mm), which improve the probability of detection (Fig. 1). Curved reformats, three-dimensional volume rendering techniques and maximum intensity projection provide important anatomic details for surgical planning (*e.g.*, tumor vascular encasement). CT scan also effectively detects nodal and metastatic disease. The majority of GEP-NETs are visible as enhanced or hypervascular lesions and are typically more conspicuous in the late arterial acquisition phases [12]. On precontrast images, these lesions are typically hypoattenuating, although some primary lesions may be small and have the same density as normal parenchyma and thus may be difficult to detect. It is therefore crucial to perform multiphasic contrast-enhanced CT, including arterial and portal venous acquisition phases. Indeed, the differences in the time elapsed after contrast administration may substantially affect image acquisition, potentially leading to both false-positive or false-negative findings that can result in an erroneous detection or response assessment. Particular caution is recommended regarding clinical decisions based on the emergence or fading of a single small lesion. The sensitivity of multidetector CT for primary pancreatic GEP-NETs ranges from 57 to 63 % and may be as high as 94 % if CT slices are reformatted in thin sections (1–1.5 mm). This detection rate substantially decreases, however, for primary extrapancreatic tumors, particularly those located in the small bowel. Some of these tumors may be only visible when mesentery involvement induces surrounding fibrosis (desmoplastic reaction) with tissue retraction. More recently, a novel modality, dual-energy CT (DECT), is an emerging technique based on imaging at two distinctly different energy levels (*e.g.*, 80 and 140 kVp) to differentiate materials. DECT can provide multiple parameters, including monochromatic CT images, iodine-based material decomposition images and spectral HU curves, and virtual unenhanced images, which may add useful tissue information for detecting GEP-NETs [13, 14].

MRI has also high spatial resolution (10–500 μm; pixel/voxel dimension >1 mm) and offers several advantages over CT scans, including the lack of ionizing radiation, superior soft tissue discrimination, and greater interobserver agreement. In addition, MRI offers the advantage of combining anatomical and functional or molecular imaging techniques, including diffusion-weighted imaging (DWI), dynamic contrast-enhanced MRI (DCE-MRI), and magnetic resonance spectroscopic imaging (MRSI). NETs are typically best observed in fat-suppressed T1-weighted sequences, such as low signal intensity lesions, and typically become hyperintense on T2-weighted sequences. Intravenous contrast enhancement is also essential for adequate characterization. Improved lesion detection and characterization have been documented with MRI for certain anatomic locations, such as the liver or pancreas. MRI is the best conventional imaging technique to detect hepatic metastases in GEP-NETs (sensitivity of 95 %) and shows a good performance for the detection of primary pancreatic NETs (sensitivity 74–94 %, specificity 78–100 %), although it is less useful than CT for the detection of primary small bowel lesions [15, 16]. However, MRI is still more expensive and time-consuming than CT and requires greater patient cooperation. These factors limit the widespread use of MRI, which is generally used as a problem-solving tool in patients with negative or equivocal findings resulting from other diagnostic procedures [9–11].

Transabdominal US is an inexpensive technique that may be used to screen solid organs in the abdomen or to direct needle biopsy for histological diagnosis (spatial resolution 50–100 μm). GEP-NETs are typically visualized as hypoechoic, well-defined masses commonly surrounded by a hyperechoic halo. The sensitivity of US for detecting GEP-NETs is, nevertheless, limited, ranging from 15 to 80 % depending on tumor size and anatomical localization [5, 9–11]. Its utility is further limited in patients with abundant abdominal gas or in very large/fat patients, as sound waves are attenuated as they pass deeper into the body. Improved image resolution can be achieved using a high-frequency endoscopic US (EUS) probe (7.5–12 MHz) that enables the transducer to maintain close proximity to target lesions. The sensitivity of this probe is substantially higher than the standard US probe (3–5 MHz) and is especially helpful for detecting small lesions in the pancreatic head or duodenal wall [17]. In fact, EUS sensitivity is higher than that of the CT scan in this context (92 *vs.* 63 %), particularly for detecting small insulinomas (84 *vs.* 32 %) [18]. EUS is, nonetheless, invasive, technically difficult, operator-dependent, and not widely available [5, 9–11]. Finally, intraoperative US (IOUS) may be an important aid in the surgical exploration of the pancreas and liver.

Finally, multimodal imaging is able to provide a combination of anatomical, molecular, and functional imaging quantitative parameters of tumor phenotype. In modern scanners, positron emission tomography (PET) and single photon emission computed tomography (SPECT) are combined with an anatomical cross-sectional counterpart such as CT (PET/CT and SPECT/CT) and also MRI (PET/MRI) [19]. These hybrid techniques have demonstrated to be useful of GEP-NETs for tumor detection, staging, and tumor response evaluation [20].

### Standard size-based evaluation criteria

The first widely adopted criteria for evaluating cancer therapies were developed by the World Health Organization (WHO) in 1979 and were primarily intended for use in clinical trials that had a tumor response as the primary endpoint [21]. The WHO criteria were the first set of rules to introduce the concept of overall assessment of tumor burden based on the sum of the bidimensional products of tumor lesions. In addition, they defined the response to therapy based on the percentual change from baseline. This standardization facilitated a common universal language for reporting the results of cancer therapy in a consistent manner to enable cross-trial comparisons. Numerous modifications of the WHO criteria were subsequently made to clarify uncertain issues in the original document and to accommodate emerging new technologies such as CT and MRI. These revisions led, paradoxically, to the response criteria being no longer comparable among research organizations. An International Working Party was thereby constituted to simplify and standardize again the evaluation criteria that resulted in the *Response Evaluation Criteria In Solid Tumors* (RECIST, version 1.0) [3]. Key features of these updated criteria included the use of one-dimensional measurements for assessing tumor burden, the definition of the minimum size of measurable lesions to be selected as targets, instructions on how many lesions to follow and how, and a model for the overall response assessment combining both target and nontarget lesions [3, 4]. In addition, time to progression and PFS, which can be assessed in all patients, as opposed to duration of response that can only be quantified in responders, were briefly discussed as alternative endpoints in certain circumstances (*i.e.*, investigation of noncytoreductive anticancer agents) [7, 8].

An updated version of RECIST (version 1.1) was published in 2009 [4] to address several questions and pending issues, including how to apply RECIST in trials in which progression, rather than response, is the primary endpoint, or in trials of targeted noncytotoxic drugs. Whether and how to use newer imaging technologies, such as ^18^F-fluorodeoxyglucose positron emission tomography (^18^F-FDG-PET) and MRI, was also addressed. A summary of the evolution from WHO response criteria to RECIST 1.1 is presented in Table 2.

**Table 2:** Evolution of tumor response criteria: from WHO to RECIST 1.1

	WHO	RECIST 1.0	RECIST 1.1
Method to assess tumor burden	Sum of products of the longest and greatest perpendicular diameters of all measured lesions (bidimensional)	Sum of longest diameters of target lesions (one-dimensional)	Sum of longest diameters for nonnodal and short axis for nodal target lesions (one-dimensional)
Definition of measurable disease	Not specified	CT: ≥10 mm with spiral CT ≥20 mm with nonspiral CT Clinical: ≥20 mm LN, not specified	CT: ≥10 mm longest diameter for nonnodal ≥15 mm short axis for nodal lesions Clinical: ≥10 mm (measured with calipers) Special considerations for bone and cystic lesions
Number of target lesions to follow	Not specified	Maximum of 10 lesions (up to 5 per organ) Should be those with longest diameters, representative of all involved organs, and most suitable for accurate repeated measurement	Maximum of 5 lesions (up to 2 per organ) Should be those with longest diameters, representative of all involved organs, and most suitable for accurate repeated measurement
Response categories			
• CR	Disappearance of all known disease, confirmed at 4 weeks	Disappearance of all known disease, confirmed at 4 weeks	Disappearance of all target and nontarget lesions LN <10 mm short axis
• PR	≥50 % decrease of tumor burden, in the absence of new lesions, confirmed at 4 weeks	≥30 % decrease of tumor burden, taking baseline sum as reference, in the absence of new lesions, confirmed at 4 weeks	≥30 % decrease in tumor burden, taking baseline sum as reference, in the absence of new lesions, to be confirmed at 4 weeks only in nonrandomized trials with response as primary endpoint
• SD	Neither sufficient shrinkage to qualify for PR nor sufficient increase to qualify for PD	Neither sufficient shrinkage to qualify for PR nor sufficient increase to qualify for PD	Neither sufficient shrinkage to qualify for PR nor sufficient increase to qualify for PD
• PD	≥25 % increase in tumor burden or appearance of new lesions	≥20 % increase in tumor burden, taking the smallest sum since treatment started as reference, or appearance of new lesions	≥20 % increase in tumor burden, taking the smallest sum since treatment started as reference, with a minimum absolute value increase ≥5 mm or appearance of new lesions

*CR* complete response, *LN* lymph nodes, *PD* progressive disease, *RECIST* Response Evaluation Criteria In Solid Tumors, *PR* partial response, *SD* stable disease, *WHO* World Health Organization

### Pitfalls of size-based response assessment in GEP-NETs

The RECIST criteria have been widely adopted by academic institutions, cooperative groups, and industry as a standard method for reporting clinical trials. The primary strengths of these criteria are simplicity, reproducibility, and universal acceptance, which allow outcomes across different trials to be compared. Nevertheless, a number of limitations remain, and several unique features of tumor biology and specific cancer therapies make their applicability particularly challenging in the field of GEP-NETs [22, 23].

RECIST dichotomize patients into responders *versus* nonresponders to quantify drug efficacy, the latter category including both stabilization of disease (which in certain scenarios may be actually indicative of a drug-induced antiproliferative effect) and disease progression. Even the latest version of RECIST primarily focuses on the use of objective response endpoints for phase II trials [24]. In the context of GEP-NETs, however, this may be only applicable to poorly differentiated, highly proliferative tumors that are treated with cytotoxic therapy. As the majority of GEP-NETs are well-differentiated, slow-growing tumors, and novel targeted agents are increasingly being used to treat this disease, alternative definitions of tumor response are being actively explored and greater emphasis is being placed on progression-based endpoints [23].

Other means for assessing treatment effects, such as a minor response (tumor shrinkage <30 %), disease control rates (a combination of objective response and stable disease), or the proportion of patients that are progression-free at landmark time points, could be considered as alternative metrics for providing early indications of an agent deserving additional clinical development. A major limitation to this approach in the field of GEP-NETs is to accurately estimate the expected disease stabilization rate, in the absence of a treatment effect, in this generally indolent disease. For this reason, a randomized controlled design is preferred, when feasible, for phase II screening trials in this context.

Several recent studies have illustrated how alternative metrics may more accurately reflect the therapeutic effect of medications in well-differentiated GEP-NETS. Indeed, two somatostatin analogs (octreotide and lanreotide) and two targeted agents (everolimus and sunitinib) significantly improve PFS of patients with different subtypes of GEP-NETs with little or no effect on tumor volume (objective response rate <10 % by RECIST) [5–8]. Waterfall plots of pivotal randomized trials, such as RADIANT-3 to evaluate everolimus in pancreatic NETs, showed that the proportion of patients achieving any degree of tumor shrinkage was substantially greater in everolimus-treated patients (64 %) than in patients in the placebo control arm (21 %), although response rates were low in both treatment arms (5 *vs.* 2 %) [8]. The current thresholds used to define tumor progression (≥20 %), which have become more conservative over time, have also been questioned in the context of slow-growing GEP-NETs. However, caution should be advised in lowering this threshold, as validation would be required to ensure that the measurement errors are actually inferior to the cutoff values, and this is not an easy task due to the technical difficulties frequently encountered in the precise definition of lesions in NETs.

Other clinical settings that question the validity of RECIST to address treatment failure include clinical or biochemical progression in the absence of radiological progression, focal progression that is amenable to local therapy (*e.g.*, small bowel obstruction with stable liver metastasis), or indolent asymptomatic progression. Some technical difficulties inherent to identifying and monitoring metastases in certain patients with GEP-NETs may also limit the application of RECIST. These include patients with small volume metastatic disease or, conversely, extensive liver involvement with either multiple small or large confluent liver metastases, which can form conglomerate masses that may be difficult to individualize and monitor. In addition, certain targeted agents (*e.g.*, angiogenesis inhibitors) may induce necrosis or cystic changes in the tumor that are not only not associated with tumor shrinkage but may even render preexisting lesions more visible, which may be misleading and erroneously interpreted as progressive disease rather than as a positive therapeutic effect. Finally, residual masses may not be adequately differentiated from fibrosis, with no viable tumor, by means of standard imaging modalities, and would never be considered as complete responses by RECIST [23].

### Alternative response evaluation criteria

Beyond size, additional radiological parameters can also provide very valuable information in terms of antitumor efficacy and are increasingly being considered when evaluating response in certain tumor types treated with specific targeted agents. Indeed, necrosis, hemorrhage, and myxoid degeneration may reflect pathologic tumor response in the absence of significant tumor shrinkage. One of the most representative examples of this effect was soon documented in gastrointestinal stromal tumors (GIST) after the introduction of imatinib. The dramatic changes in tumor density induced by this drug, as determined by measuring CT attenuation coefficient in Hounsfield units (HU), led Choi to propose in 2007 a new set of criteria that combined changes in both size and density for tumor response assessment in this setting [25] (Table 3). These criteria and their variants are now widely applied in GIST and are being prospectively evaluated in other neoplastic diseases. Preliminary data in patients with pancreatic NETs suggest that Choi criteria may help to early discriminate patients who might benefit from sunitinib or everolimus therapy [26].

**Table 3:** Alternative functional tumor response criteria

Response categories	Response criteria			
	Choi	mRECIST	MASS	PERCIST
CR	Disappearance of all lesions and no new lesions	Disappearance of any intratumor arterial enhancement in all target lesions No new lesions	*Favorable response* ≥20 % decrease in tumor burden per RECIST or ≥10 % decrease in tumor burden per RECIST and ≥ half of nonlung target lesions with a ≥20 HU decreased mean attenuation or One or more nonlung target lesions with a ≥40 HU decreased mean attenuation No new lesions	Complete resolution of ^18^F-FDG uptake within tumor volume so that it is less than mean liver activity and indistinguishable from surrounding background blood-pool levels
PR	≥10 % decrease in tumor burden per RECIST or ≥15 % decrease in tumor density (HU) on CT scan and no new lesions nor unequivocal progression of nonmeasurable disease	≥30 % decrease in tumor burden per RECIST considering only viable tumor of target lesions (that with arterial enhancement on CE radiological techniques) No new lesions		≥30 % relative and ≥0.8 absolute decrease in ^18^F-FDG uptake (SUL peak of target lesion) and no >30 % increase in SUL of nontarget lesions and no PD by RECIST ROI does not need to be in precise same area as baseline scan
SD	Does not meet criteria for complete, partial, nor progressive disease and No clinical deterioration attributable to tumor progression	<30 % decrease to ≤20 % increase in the sum of maximum arterial enhancing diameter of target lesions No new lesions	*Indeterminate response* Does not fulfill criteria for favorable or unfavorable response No new lesions	Does not fulfill criteria for partial response nor for progressive disease
PD	≥10 % increase in tumor size per RECIST that does not meet criteria for PR by tumor density on CT scan or Appearance of new lesions including new intratumor nodules or increase in size of existing nodules	>20 % increase in tumor burden per RECIST considering only viable tumor of target lesions or Appearance of new lesions	*Unfavorable response* ≥20 % increase in tumor burden per RECIST or Target lesion with central necrosis changing to near complete enhanced solid tumor or New enhancement in a nonenhancing lesion or Appearance of new lesions	>30 % relative and 0.8 absolute increase in ^18^F-FDG uptake (SUL peak of target lesion) or Unequivocal increase in extent of ^18^F-FDG uptake (75 % in total lesion glycolysis volume with no decline in SUL) or New ^18^F-FDG-avid lesions

^*18*^ *F-FDG* ^18^F-fluorodeoxyglucose, *CT* computed tomography, *EASL* European Association for Study of the Liver, *EORTC* European Organization for Research and Treatment of Cancer, *HU* Hounsfield unit, *MASS* morphology, attenuation, size and structure, *mRECIST* modified RECIST, *PERCIST* Positron Emission Response Criteria In Solid Tumors, *PET* positron emission tomography, *RECIST* Response Evaluation Criteria In Solid Tumors, *ROI* region of interest, *SUL* standard uptake value

Other alternative methods to evaluate tumor response (Table 3) have been described in detail elsewhere [27–30] and are beyond the scope of this review. Briefly, these methods take into consideration additional parameters such as arterial enhancement (*e.g.*, the *European Association for Study of the Liver* (EASL) criteria in hepatocellular carcinoma) [27], treatment-induced tumor necrosis, or other structural changes (*e.g.*, the Morphology, Attenuation, Size and Structure (MASS) criteria for renal cancer treated with antiangiogenic agents, or the Lee criteria for nonsmall cell lung cancer treated with EGFR inhibitors) [31, 32]. Specific criteria have also been developed for novel therapeutic strategies (*e.g.*, immune-related response criteria) [33] or imaging modalities such as PET (*e.g.*, PET response criteria in solid tumors or PERCIST) [30, 34, 35].

^18^FDG-PET assesses tumor glucose uptake, which broadly correlates with cancer cell viability. ^18^FDG uptake may therefore provide an early and sensitive pharmacodynamic marker for monitoring response to antiproliferative agents [36]. Changes in ^18^FDG uptake measured by PET have been correlated with a pathological tumor response and overall survival in certain neoplastic diseases. Caution should be advised, nevertheless, as ^18^FDG-PET is a sensitive but nonspecific method for detecting malignancy sites. Areas of active inflammation or infection are common sources of false-positive findings [37]. A period of at least 10 days (or preferably up to 3 weeks) is advised before a PET scan is performed following chemotherapy administration, to bypass transient fluctuations of ^18^FDG uptake, either stunning or flare of tumor uptake, which may occur soon after treatment. False-negative findings may also arise when evaluating small lesions. PET imaging is progressively being incorporated for staging and response assessment in a number of malignancies (*e.g.*, lymphoma) [38]. In the context of GEP-NETs, however, it would be only applicable to highly proliferative or poorly differentiated tumors, as guidelines have been specifically developed for the ^18^FDG radionuclide. Whether these criteria may be applied to other radiotracers (*e.g.*, ^68^Gadolinium-tetraazacyclododecane tetraacetic acid (^68^Ga-DOTA) peptides) remains to be elucidated. Nevertheless, prospective trials for validating these response criteria are warranted.

## New imaging modalities for assessing GEP-NETs

### Cancer-specific functional imaging

Functional imaging refers to the visualization, characterization, and quantification of biological processes at the cellular or molecular level. The unique features of GEP-NETs provide distinct targets for cancer-specific functional imaging, such as somatostatin receptors (SSTR), or catecholamine transporter and synthesis pathways. Consequently, an increasing number of radiopharmaceuticals are becoming available to detect and quantify different aspects of the heterogeneous biology of GEP-NETs (Table 1). Several techniques that are increasingly being used in clinical practice will be discussed below.

#### Somatostatin receptor imaging: SRS and ^68^Ga-DOTA peptides

**Fig. 2:**
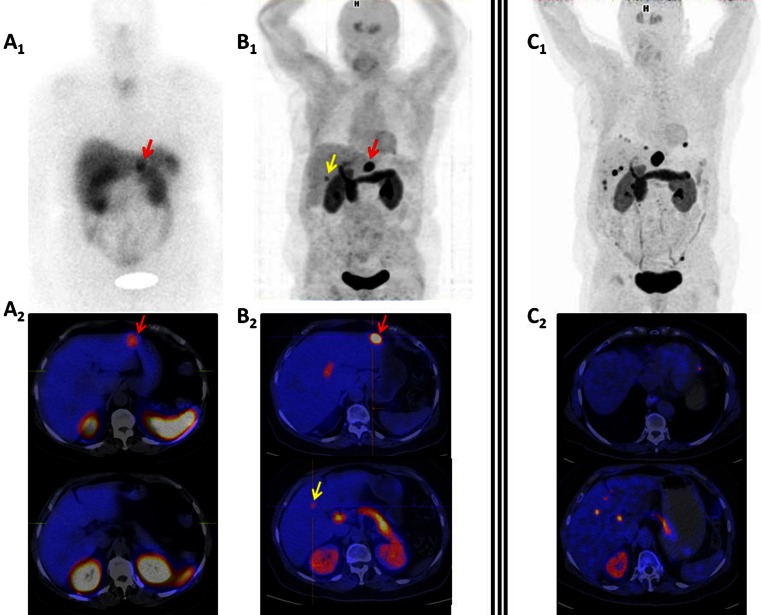
SRS using ^111^In-pentetreotide (*a*) and ^18^F-FDOPA PET/CT (*b*) performed 1 week apart in a patient with a well-differentiated metastatic ileal NET. Planar scintigraphy (*a1*) shows an uncertain liver lesion (*red arrow*), clearly located in segment II of the liver in the SPECT/CT fusion image (*a2*). Whole-body PET/CT image using ^18^F-FDOPA (*b1* and *b2*) shows the same liver metastasis (*red arrow*) but also detects an additional liver lesion in segment V (*yellow arrow*). Follow-up ^18^F-FDOPA PET/CT (*c1* and *c2*) performed after 12 cycles of octreotide therapy identifies multiple hepatic and peritoneal implants (both abdominal and subdiaphragmatic costophrenic angle) reflecting tumor progression

Scintigraphy with radiolabeled somatostatin analogs (SRS) is a very useful imaging technique for detecting tumors expressing somatostatin receptors. ^111^In-DTPA-D-Phe^1^-octreotide (^111^In-pentetreotide), a metabolically stable radiopharmaceutical with a high affinity for type 2 somatostatin receptors (SSTR2), is the most commonly used tracer for imaging GEP-NETs [39–41]. The major limitations of SRS are the evaluation of organs with higher physiological uptake (*e.g.*, liver and gut) and the detection of small lesions owing to its low spatial resolution (range 7–15 mm) [42]. The addition of SPECT to SRS planar views and, more recently, the multimodal devices that combine SPECT and computed tomography (SPECT/CT) have led to improved tumor detection. These approaches enable a more precise identification of the physiological uptake of the radiolabeled analog and a better definition of the functional significance of lesions detected by CT (Fig. 2) [43, 44].

Next-generation somatostatin analogs have been developed using the chelator DOTA rather than diethylenetriaminepentacetate (DTPA), and these molecules can also be labeled using positron-emitting radionuclides, such as ^68^Ga (Table 1). ^68^Ga-DOTA-Tyr^3^-octreotide (^68^Ga-DOTA-TOC), ^68^Ga-DOTA-Tyr^3^-octreotate (^68^Ga-DOTA-TATE), and ^68^Ga-DOTA-1-NaI^3^-octreotide (^68^Ga-DOTA-NOC) [45] add higher SSTR affinity and more favorable pharmacokinetic properties (namely, more rapid extraction and clearance) to the improved spatial resolution of PET relative to SRS (range 4–10 mm). ^68^Ga-labeled somatostatin receptor PET (SRPET) is in fact increasingly being used in specialized centers and will replace SRS in the near future. Indeed, a recent meta-analysis has shown that SRPET has a higher diagnostic accuracy (sensitivity, 93 %; specificity, 96 %) than that reported for SRS (sensitivity, 82–95 %; specificity, 50–80 %) [46]. Additional advantages of SRPET include the short scanning time required, the relatively low radiation exposure, and the availability of ^68^Ga generators.

Sensitivity may substantially vary depending on the tumor type and the specific binding and affinity profile of the radiopeptide used [47]. SSTR2A and SSTR5 are the most frequently expressed SSTRs in GEP-NETs (86 and 62 %, respectively), followed by SSTR1 [48–50]. Nevertheless, SSTR2A expression varies significantly among different GEP-NETs and is much less frequent in insulinomas (58 %) than in gastrinomas (100 %) or carcinoid tumors (86 %). Overall, well-differentiated neoplasms have a higher density and more homogeneous distribution of SSTRs than poorly differentiated endocrine carcinomas, except for SSTR5 that shows the opposite trend. SSTR expression is generally lower in pancreatic than in gastrointestinal tumors except SSTR3, which has greater expression in pancreatic than in enteric NETs (40 *vs*. 21 %). On the other hand, SRS using ^111^In-pentetreotide has high affinity for SSTR2, and consistently, comparative analyses have revealed a high correlation between tumor detection using SRS and SSTR2A expression assessed by immunohistochemistry (Table 2). However, ^68^Ga-labeled DOTA peptides show superior affinity for SSTR2 than ^111^In-pentetreotide. ^68^Ga-DOTA-TATE has the highest affinity for SSTR2, but only ^68^Ga-DOTA-NOC shows high affinity for SSTR3 and SSTR5 (Table 4). Concordant with these affinity profiles, tissue immunoreactive scores for SSTR2A and SSTR5 correlate with SUV values on PET/CT using ^68^Ga-DOTA-NOC [50], and SSTR2A correlates with ^68^Ga-DOTA-TOC [51]. Such differences should be considered in the clinical setting because sensitivity may be lower with ^111^In-pentetreotide SRS or ^68^Ga-DOTA-TATE SRPET for certain tumor types, such as insulinomas, whereas ^68^Ga-DOTA-NOC may be more suitable for pancreatic NETs [49].

**Table 4:** Radiopeptide affinity (IC-50 values in nmol/L) profile for somatostatin receptors (SSTR) commonly expressed in NETs

	SSTR2A (nmol/L)	SSTR3 (nmol/L)	SSTR4 (nmol/L)	SSTR5 (nmol/L)
SRS				
^111^In-pentetreotide	22	–	–	–
SRPET				
^68^Ga-DOTA-TOC	2.5	–	–	73
^68^Ga-DOTA-TATE	0.2	–	–	–
^68^Ga-DOTA-NOC	1.9	40	–	7.2

*SSTR* somatostatin receptor, *SRS* somatostatin receptor scintigraphy, *SRPET* somatostatin receptor positron emission tomography, *−* low or absence of affinity

**Fig. 3:**
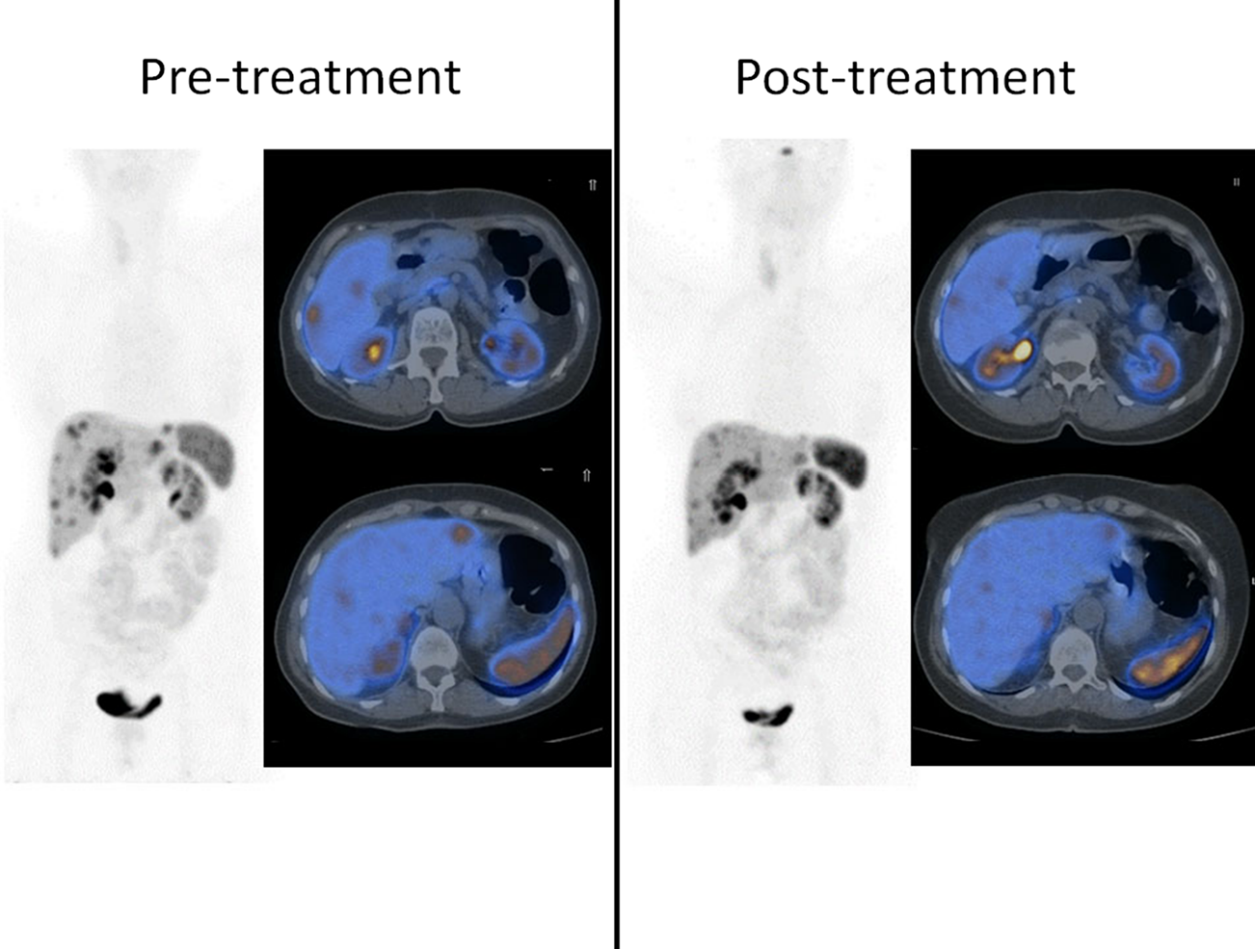
SRPET using ^68^Ga-DOTANOC in a patient with multiple liver metastases of a well-differentiated ileal NET. PET/CT scans performed before (*left*) and after 4 cycles of PRRT (^177^Lu-DOTA-TATE) and subcutaneous monthly lanreotide (*right*) show a partial response to therapy (courtesy of Valentina Ambrosini and Stefano Fanti, S. Orsola-Malpighi University Hospital, Bologna, Italy)

The clinical applications of these new imaging modalities are likely to expand because novel somatostatin analogs are being developed with increased affinity for different SSTR subtypes. For example, whereas octreotide and lanreotide have high affinity for SSTR2, pasireotide, a new somatostatin analog not approved for the treatment of NETs, has a higher affinity for the remaining SSTRs, especially SSTR5. In this context, future studies should address the potential correlation between specific SSTR tumor expression profiles assessed by immunohistochemistry and molecular imaging and response to SSTR-targeted therapy [50] (Fig. 3). Nevertheless, poorly differentiated GEP-NETs are more suited to be characterized using the most widely available radiotracer (^18^F-FDG), which reflects the increased glucose transport of rapidly proliferating cells. Indeed, the sensitivity of ^18^F-FDG PET for depicting GEP-NETs with high proliferative indices (Ki-67 >15 %) exceeds that of SRS (92 *vs.* 69 %), and ^18^F-FDG SUV uptake is a stronger prognostic factor in this context than traditional biochemical or histological markers, such as chromogranin A (CgA) or Ki-67 index [40, 41].

Procedure guidelines for SRS and SRPET tumor imaging by the *European Association of Nuclear Medicine* (EANM) and *European Neuroendocrine Tumors Society* (ENETS) have included monitoring of response to different therapies in their clinical indications [42, 52, 53]. With the emergence of PRRT as a novel treatment strategy for GEP-NETs, molecular imaging has gained relevance as a potential tool for therapy response assessment. SSTR tumor expression documented by SRS and, more recently, SRPET is a prerequisite for PRRT planning in GEP-NETs [54]. Initial reports failed to show an additional advantage of ^68^Ga-DOTA-TOC PET/CT over conventional radiologic imaging (CT or MRI) in evaluating response to PRRT, except for an earlier detection of metastatic disease in a subgroup of patients using SRPET [55]. In contrast, a transient decrease in ^68^Ga-DOTA-TATE tumor uptake after the first cycle of PRRT was predictive of time to progression and symptom relief in patients with GEP-NETs, with ΔSUV_T/S_ (tumor/spleen SUV ratio) being superior to ΔSUV_max_ for prediction of outcome [56]. Notably, the lack of SSTR expression assessed by ^68^Ga-labeled PET imaging and the documentation of hypermetabolism by ^18^F-FDG PET have been associated with rapid progression and poor prognosis in GEP-NET patients treated with both PRRT and watchful waiting follow-up strategies, suggesting that these molecular imaging techniques also characterize tumor biology independently of therapy [57, 58].

More recent reports have correlated baseline (pretherapeutic) ^68^Ga-PET SUV values with the subsequent absorbed dose of radiopharmaceuticals and the clinical outcome after PRRT, both with ^177^Lu-DOTA-TATE and ^90^Y-DOTA-TOC. Moreover, the mean per-cycle decrease of tumor-absorbed dose was linked to the morphologic response following treatment with ^177^Lu-DOTA-TATE, suggesting that sequential changes of tumor-absorbed doses could be appropriate early markers of therapeutic response. These observations reinforce the potential role of SRPET for the prediction of outcome after PRRT, providing the rationale for individual dosing and allowing a more appropriate selection of patients who might benefit from this therapeutic strategy [59–62]. Additional larger and prospective studies should be performed, nonetheless, to ascertain these correlations and to standardize and validate appropriate cutoff values of quantitative molecular parameters before these techniques can be widely used for clinical decision-making. The possibility of tumor dedifferentiation with loss of SSTRs should also be considered as an additional limitation to SRPET, although it may be overcome by using different radiotracers (*e.g.*, ^18^F-FDG). Finally, molecular imaging could also have a role in evaluating response to treatment modalities other than PRRT, including cytotoxic agents or “cold” targeted agents; however, this has not been formally assessed to date.

In summary, the lack of spatial resolution and detectability of conventional SRS has been partially overcome using multimodal tomographic techniques (SPECT/CT). However, the advent of new devices with higher spatial resolution such as PET/CT or even PET/MRI using new generation radiotracers provides the highest available sensitivity (detection of radiotracer concentration in tissue at the nanomolar range). These advantages might therefore compensate some of the limitations that traditionally have been ascribed to nuclear medicine techniques. Standardization and validation of these novel techniques and assessment criteria in prospective clinical trials, to ensure results are reliable and reproducible, are essential however before their widespread use in everyday clinical practice.

#### Catecholamine transporter and synthesis: ^18^F-DOPA and ^11^C-5-HTP

Alternative molecular imaging techniques exploit the intrinsic property of neuroendocrine cells for amine precursor uptake and decarboxylation (the APUD system). Several precursors, such as 5-hydroxy-L-tryptophan (5-HTP) and L-dihydroxyphenylalanine (L-DOPA), are taken up by neuroendocrine cells and converted to serotonin and dopamine, respectively. These precursors can be labeled to produce PET tracers that are useful for GEP-NET imaging. The most widely available marker is ^18^F-fluoro-L-3,4-dihydroxyphenylalanine (^18^F-DOPA), which enters the neuroendocrine cells *via* an L-type amino acid sodium-independent transporter (LAT). There, it is subsequently decarboxylated by the aromatic L-amino acid decarboxylase, an enzyme highly expressed in neuroendocrine cells, resulting in ^18^F-dopamine. Then, this molecule is transported into cytoplasmic storage secretory vesicles and protected from enzymatic degradation, thereby promoting its intracellular retention. A high ^18^F-DOPA uptake is commonly observed in neuroendocrine tumor cells, particularly in small-bowel serotonin-producing NETs [63].

^18^F-DOPA has greater sensitivity for GEP-NET detection than SRS (Fig. 2), even when SPECT/CT is used [64]; however, comparative studies between ^18^F-DOPA and ^68^Ga-DOTA peptides tilt the balance in favor of SRPET in terms of diagnostic accuracy [65]. In this regard, it is important to note the lack of correlation between ^18^F-DOPA transport and decarboxylation and the expression of SSTR depicted by SRPET [66]. Thus, ^18^F-DOPA PET/CT is generally recommended for GEP-NET diagnosis when conventional radiological imaging and SRS or even SRPET show negative or inconclusive findings.

^18^F-DOPA PET, as a molecular imaging tool that reflects cellular metabolic activity rather than receptor density, has been postulated to be likely a more appropriate tool to monitor treatment response. Supporting this hypothesis, ^18^F-DOPA uptake is frequently increased in GEP-NET patients with elevated plasma serotonin, and whole-body metabolic tumor burden (WBMTB) assessed using ^18^F-DOPA PET is correlated with urinary and plasma levels of tumor markers belonging to the serotonin and catecholamine pathways [66, 67]. Interestingly, urinary excretion of 5-hydroxyindoleacetic acid (5-HIAA), the primary metabolite of serotonin, is also a reliable indicator of tumor burden and metabolic activity and one of the most widely used markers for response assessment and follow-up of patients with functioning GEP-NETs. WBMTB could therefore potentially become an alternative parameter for evaluating disease extent, biochemical activity, and tumor response in these patients.

The ^11^C-5-hydroxy-L-tryptophan (^11^C-5-HTP) PET tracer, a precursor of serotonin, is a useful universal imaging tool for detecting GEP-NETs, with greater sensitivity than CT or SRS. ^11^C-5-HTP PET seems to perform better than ^18^F-DOPA for the overall detection of GEP-NETs; but unlike ^18^F-DOPA, its use is restricted to a few specialized centers as the ^11^C radioisotope has a very short half-life. This radiopharmaceutical is generally used, if available, when conventional imaging procedures fail to locate an occult tumor, or for follow-up purposes when clinical, biochemical, and standard radiological assessments are equivocal or show conflicting results [68]. A close correlation between changes in ^11^C-5-HTP transport rate and urinary 5-HIAA excretion during medical treatment indicates its potential as a means for monitoring treatment efficacy [69]. However, besides anecdotal case reports, studies assessing the value of serial changes in ^18^F-DOPA or ^11^C-5-HTP PET metabolic parameters for evaluating response to therapy are still lacking.

Other NET-specific radiolabeled peptides, currently in preclinical or early clinical development, include glucagon-like peptide-1 (GLP-1), cholecystokinin (CCK), gastrin, bombesin, substance P, vasoactive intestinal peptide (VIP), and neuropeptide (NP)-Y analogs. Additional noncancer-specific tracers of potential use in GEP-NETs include ^18^F-fluoro-L-thymidine (^18^FLT)-PET, a nonspecific marker of proliferation, and ^18^F-fluoro-misonidazole (^18^FMISO)-PET, which accumulates in hypoxic tissues and could hence be a potential biomarker for assessing response to antiangiogenic therapy.

### Therapy-specific functional imaging

As treatment options for GEP-NETs continue to expand, evaluation of therapeutic response solely on the basis of size clearly has substantial limitations, particularly in the era of targeted therapy. Indeed, new therapeutic modalities, such as angiogenesis inhibitors, may significantly increase patients’ survival by inducing a cytostatic effect that does not necessarily translate into tumor shrinkage (*e.g.*, tumor necrosis or cavitation with no size reduction). Functional imaging techniques that monitor specific physiological and cellular processes within the tumors in response to antineoplastic agents with novel mechanisms of action are therefore gaining momentum [22, 70–74]. In fact, these alternative response assessment techniques are increasingly being implemented at earlier stages of drug development. The main features of these imaging modalities are summarized in Table 1.

**Fig. 4:**
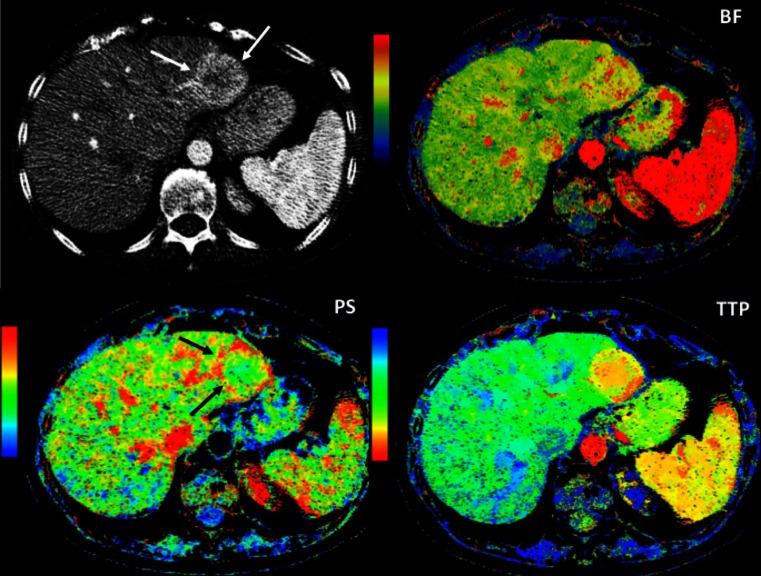
Perfusion CT images in a patient with NET liver metastases. Conventional CT image depicts a hypervascular liver metastasis in the left liver lobe (*white arrows*). Parametric maps of blood flow (*BF*), permeability (*PS*), and time to peak (*TTP*) show a different functional behavior at the periphery of the metastatic deposit (*black arrows*) compared to normal liver or the center of the metastasis, with increasing BF and PS and decreasing TTP. Perfusion CT provides additional information compared to morphologic imaging that may have prognostic value or be useful in tumor response evaluation

DCE imaging methods may be applied to US, CT, or MRI with contrast dyes specific to each modality. These imaging techniques assess tumors based on the appreciable differences between the heterogeneous, chaotic, and leaky neoplastic vascular network and the normal physiological vasculature of healthy tissues [22, 74]. A rapid sequence of images is acquired through a volume of interest before, during, and after the intravenous administration of contrast material. These data are then fitted to mathematical models to analyze a number of physiological processes and to obtain quantitative perfusion parameters that reflect the vascular characteristics of the examined tissue, including blood volume (BV), blood flow (BF), mean transit time, and permeability area. Although experience is limited, DCE-US, DCE-CT, and DCE-MRI are likely to become useful tools for characterizing GEP-NETs [75, 76] and to evaluate their biologic aggressiveness [77], therapeutic response [22, 74] (Fig. 4), and prognosis [78, 79].

DCE-US enables the quantification of perfusion parameters by injecting ultrasonic microbubble-based contrast dye, and international guidelines have been produced to standardize this technique [80]. Among these parameters, the area under the perfusion curve (AUC)—a criterion linked to BV—has been identified as a reliable early predictor of response to antiangiogenic therapy using the RECIST criteria [81]. This finding was further validated in a multi-institutional cohort of 539 patients with various tumor types, including GEP-NETs, treated with different antiangiogenic agents [82]. In this group, early decreases in perfusion parameters were significantly associated with improved time to disease progression and overall survival. DCE-US has also been tested in a small study of patients with GEP-NETs who were treated with transarterial embolization (*n* = 10) or chemoembolization (*n* = 7). The authors proposed a new composite parameter combining functional and morphological data, named tumor vitality index, which may warrant additional exploration [83]. DCE-US allows early identification of tumor structural changes in response to PRRT, as decreased perfusion parameters are documented as soon as 6 weeks after therapy, whereas morphological changes may require a minimum of 6 months follow-up to be detectable [84]. DCE-US yielded comparable results to DCE-CT in the assessment of perfusion and morphological changes of liver metastases, particularly lesions located in the caudal and ventral parts of the liver, although adequate visualization of subdiaphragmatic structures remains an important limitation of this technique.

**Fig. 5:**
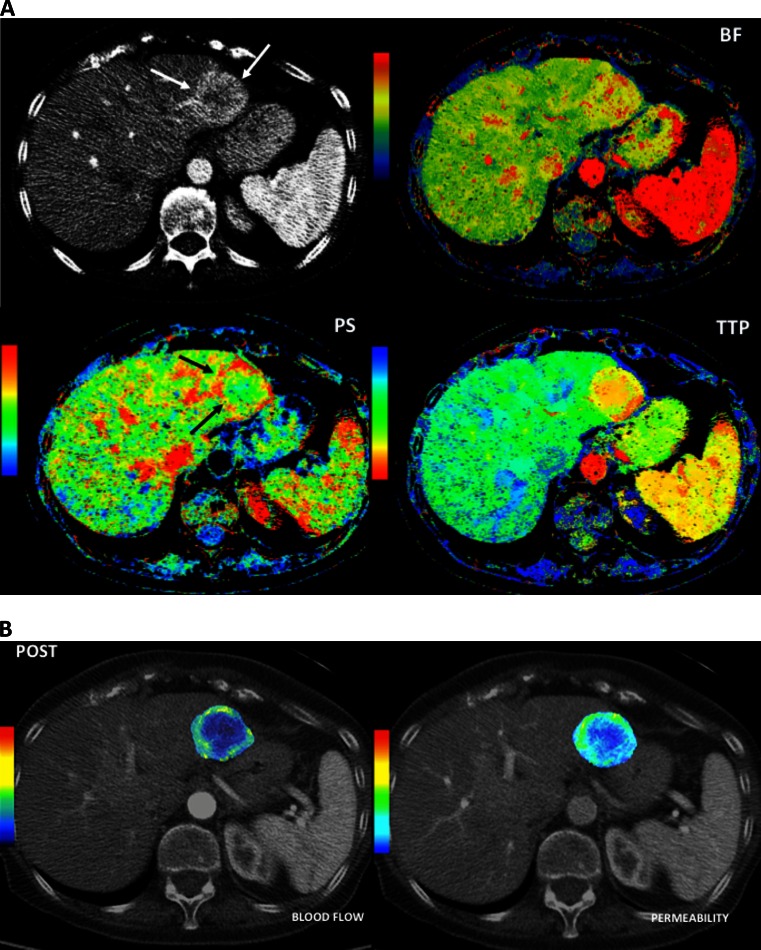
Perfusion CT images in a patient with liver metastases from a neuroendocrine tumor pre- and posttherapy using antiangiogenic drugs. Parametric maps of blood flow and permeability superimposed over conventional CT images. Pretherapy study (**a**) demonstrated increased mean values of blood flow (115 mL/min/100 g) and permeability (51 mL/min/100 g), mainly at the periphery of the metastatic deposit. Posttherapy exam (**b**) evidenced a clear tumor response with drastic decrease of the values of both parameters (blood flow = 12 mL/min/100 g and permeability = 12 mL/min/100 g)

DCE-CT, also called perfusion CT, similarly enables the quantification of perfusion parameters to characterize tumor vascular features without some of the limitations of DCE-US (Fig. 4). The main drawbacks of this approach are the lack of standardization for data interpretation and the high radiation dose required*.* A significant correlation between tumor BF assessed by DCE-CT and histological assessment of intratumor microvessel density has been reported in pancreatic NETs [78]. Of note, a higher BF was observed in tumors with lower proliferation indexes, less aggressive histological features, and improved prognosis, as opposed to what may be found in other malignancies [78, 85]. DCE-CT has successfully been used to assess tumor perfusion changes in response to therapy in a small randomized phase II study comparing two antiangiogenic therapies, bevacizumab *versus* pegylated-interferon alpha 2b, in patients with advanced carcinoid tumors [86, 87]. A significant decrease in BF and BV was early observed (day 2 perfusion CT) compared with baseline data in bevacizumab-treated patients, but not in patients treated with interferon. Decrease in tumor BF following bevacizumab therapy was proportional to baseline BF suggesting bevacizumab decreased BF by a fixed percentage. Bevacizumab also induced objective responses by RECIST (18 *vs.* 0 % in bevacizumab *vs.* interferon arm) and was associated with longer PFS than the control arm. A subsequent study by the same group randomized 39 patients with low- to intermediate-grade NETs to receive bevacizumab or everolimus for one 21-day cycle, adding the alternate agent on cycle 2 (bevacizumab + everolimus). Serial functional CT assessments were mandatory. Bevacizumab significantly decreased tumor BF (44 %, *p* < 0.0001), and the addition of everolimus to bevacizumab was associated with further decrease in BF (29 %, *p* = 0.02). Everolimus alone was associated with 13 % increase in mean transit time (*p* = 0.02). Notably, several perfusion parameters (pretreatment tumor permeability surface, posttreatment mean transit time, percent reduction in BF, and percent reduction in blood volume) were significantly associated with best percent reduction in tumor diameters [88]. Taken together, these data suggest that perfusion CT parameters could potentially become useful surrogate markers for early response assessment to novel angiogenesis inhibitors in NETs. Figure 5 illustrates an example of tumor perfusion changes assessed by DCE-CT induced by antiangiogenic therapy. DECT provides a new parameter, the iodine uptake, which is assumed to reflect vital tumor burden by measuring the iodine uptake of active tumor. Iodinated contrast medium in lesions is mainly related to blood perfusion of viable tumor. This biological-related information could be a promising tool for evaluating tumor response [89] and would be of particular interest to assess response to antiangiogenic agents. However, to our knowledge, there are no published data concerning the use of this technique in GEP-NETs.

DCE-MRI allows the calculation of quantitative perfusion parameters by using a rapid series of T1-weighted images to observe the passage of contrast media intravascularly and as it leaks into the extravascular space. These pharmacokinetic parameters include the volume of extravascular space, the transfer constant (*K*^trans^), and the constant of return (*k*_ep_), some of which are of difficult physiological interpretation. *K*^trans^, for example, is a parameter that depends on the equilibrium between BF and the vascular network permeability. Low *k*_ep_ and *K*^trans^ values can indicate low perfusion, low permeability, and/or a small blood vessel surface area. Thus, any observed reductions in these parameters would reflect decreased blood flow and permeability in tumor lesions, which would be an indicator of therapeutic success. Miyazaki *et al.* [79] found that a lower pretreatment distribution volume and a high arterial flow fraction on baseline DCE-MRI were associated with a better response to treatment with radiolabeled octreotide therapy in patients with GEP-NET liver metastasis. Moreover, tumor and whole-liver distribution volume significantly increased after treatment in responding patients, suggesting that DCE-MRI could be a useful tool for predicting and monitoring response to PRRT in these patients. By contrast, baseline radiological patterns assessed by conventional imaging procedures (CT or MRI) did not predict response to yttrium-90 radioembolization in patients with GEP-NET liver metastasis [90]. However, DCE-MRI has poor intrapatient reproducibility, particularly in liver metastases [91]. Finally, one major limitation common to all DCE-based imaging modalities is that they only assess one or a few selected targets, which may not adequately represent the global tumor behavior, particularly in a setting of frequent tumor heterogeneity.

Another functional imaging technique increasingly being used is diffusion-weighted MRI (DW-MRI), which is based on the microscopic mobility of water molecules owing to thermal agitation. Water diffusion is basically restricted by interactions with cell membranes and macromolecules, and there is an inverse correlation between the degree of water motion and tissue cellularity and cell membrane integrity [70]. Thus, DW-MRI provides insight into cellular architecture at the millimeter scale, through a quantitative measurement of water diffusivity, called the apparent diffusion coefficient (ADC). The biological premise is that malignant tissues generally demonstrate higher cellularity, tissue disorganization, and increased extracellular space tortuosity, all of which contribute to a reduced motion of water, resulting in lower ADC values in malignant tumors compared with normal tissues [70]. The diagnostic superiority of DW-MRI over morphological techniques in a wide range of malignancies has led to the implementation of this fast sequence in all MRI exams in routine clinical practice.

**Fig. 6:**
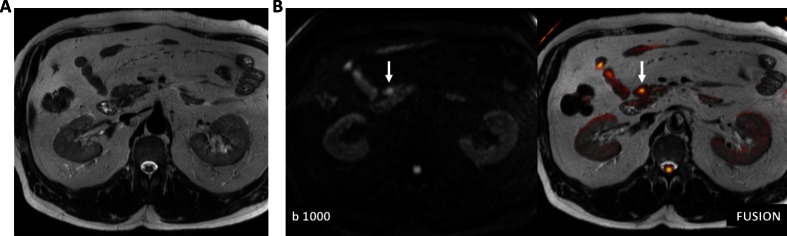
Diffusion-weighted MRI (DW-MRI) of the pancreas. **a** Axial HASTE T2-weighted image does not depict any abnormality in the uncinate process of the pancreas. **b** DW image (*left*) at high *b* value (*b* = 1000 s/mm^2^) and fused image (*right*) superimposing axial T2-weighted MRI image and color-coded map derived from high *b* value (*b* = 1000 s/mm^2^) DW image clearly demonstrate a small pancreatic neuroendocrine tumor (*arrows*) with restricted diffusion at this level

DW-MRI is clinically useful at all stages in patients with GEP-NETs, including detection (Fig. 6), tumor characterization, staging, and therapy response assessment. The fusion of DW images with high *b* value (a factor that reflects the strength and timing of the gradients used to generate DW images) and T2-weighted MRI images improves the identification of pancreatic NETs [92, 93], especially in patients with small isointense lesions observed on conventional MRI sequences [94]. Moreover, DW-MRI and ADC maps can provide information that is useful for differentiating typical and atypical hemangiomas from other hypervascular liver lesions, including GEP-NET metastases [95]. An emerging clinical application of DW-MRI is the whole-body diffusion (WBD) technique for evaluating the extent of disease. A comparative study of ^68^Ga-PET/CT *versus* WBD showed the overall superiority of ^68^Ga-PET/CT for patient staging, particularly for detecting lymph node and lung tumor deposits, whereas WBD was more accurate in detecting liver and bone metastases [96]. DW-MRI is also a valuable tool for assessing tumor aggressiveness [97, 98]. As an example, Wang *et al.* [97] found a significant inverse correlation between ADC values and tumor cellularity or Ki-67 proliferative index, and this may thus help to predict the growth rate of endocrine tumors.

Regarding the potential role of DW-MRI for predicting or monitoring response to therapy, it is important to note that changes observed in functional imaging appearance may substantially differ depending on the specific mechanism of action of each treatment modality and the effect it induces in tumors [71]. Treatments that cause apoptosis (*e.g.*, chemotherapy) result in transitory increased ADC values because of cell swelling, tumor lysis, and necrosis, although ADC values may eventually decrease as a result of tissue dehydration and fibrosis following cell death. By contrast, antiangiogenic therapies induce an initial and transient decrease in ADC values probably owing to reductions in tumor perfusion and in the extravascular-extracellular space resulting from vascular normalization and decreased vascular permeability [99]. Nevertheless, the opposite effect (ADC increase) may be observed if significant tumor necrosis is induced by vascular-targeted therapies. The complexity of interpreting DW-MRI changes induced by treatment is illustrated in a study conducted by Wulfert *et al.* [100]. In this work, DW-MRI and ^68^Ga-DOTA-TOC-PET/CT images were acquired before and 3 months after one to two cycles of intra-arterial ^90^Y-/^177^Lu-DOTA-TOC therapy in 14 patients with hepatic metastases from GEP-NET. High baseline SUV_max_ values in ^68^Ga-DOTA-TOC-PET and ADC mean values in DW-MRI were both associated with improved response to PRRT. A decrease in SUV_max_ in ^68^Ga-PET was observed only in responding lesions after one to two cycles of therapy. In contrast, mean ADC values significantly increased after treatment in both responding and nonresponding lesions. Nevertheless, nonresponding lesions with increased ADC values on first follow-up assessment were more likely to achieve a decrease in size with longer follow-up.

Locoregional therapies, such as transarterial chemoembolization, may also cause an early reduction in ADC values after therapy (within the first few hours), after which consistent increases in ADC values usually occur, coinciding with the development of cystic and necrotic changes [99, 101]. In the field of GEP-NETs, Gowdra Halappa *et al.* [102] showed a significant increase in mean volumetric ADC (27 %, *p* < 0.0001) in all patients with neuroendocrine liver metastases receiving intra-arterial therapy (chemo- or radioembolization), 3–4 weeks after therapy. In this study, a significant response assessed by DW-MRI (defined as an increase of at least 15 % in volumetric ADC) or by DCE-MRI (defined as a decrease of at least 25 % in volumetric enhancement in the arterial phase or a decrease of at least 50 % in volumetric enhancement in the venous phase) correlated with improved survival. By contrast, the response assessed using RECIST, mRECIST, and EASL criteria did not correlate with survival. Patients who presented a response only by DW-MRI (ADC) or DCE-MRI (volumetric tumor enhancement) had a similar outcome in terms of survival than those who showed a response using both imaging modalities [102].

Functional imaging is consequently a promising tool for evaluating GEP-NETs. Most studies performed to date, however, have been retrospective and have involved small heterogeneous patient populations. Important efforts for improving technical qualification and standardization are certainly warranted before any of the parameters evaluated by functional imaging can be accepted as surrogate biomarkers for response assessment in standard practice. Although these techniques can be performed on standard clinical systems, they require strict protocols, careful acquisition, accurate contrast-agent dosing and injection rate, image timing, and image analysis for quantification. The standardization of these issues is essential to guarantee reproducibility [103]. The time course and the type of tumor changes induced must be further defined for the entire range of individual therapies or treatment strategies, as well as the magnitude of treatment-induced effects that actually result in patient benefit. Large prospective validation studies must also be conducted before these techniques can assist clinicians in decision-making in current clinical practice.

## Conclusions and future perspectives

As molecular pathways governing NET cancer development and progression are being unraveled, and new anticancer agents targeting specific genomic abnormalities continue to expand, criteria and technology employed to evaluate drug antitumor activity need to evolve to accurately assess tumor response and adequately address whether individual patients benefit or not from specific therapies. Significant advances in molecular and functional imaging techniques have provided new evaluation parameters that may potentially improve response assessment to novel therapeutic approaches. These endeavors are particularly relevant in the field of NETs, commonly slow-growing tumors in which major tumor shrinkages are unlikely to happen. Optimization of these measures to monitor response and anticipate the emergence of tumor resistance leading to uncontrolled tumor growth is a major focus of research. Standardization and validation of these novel techniques and assessment criteria in prospective clinical trials, to ensure results are reliable and reproducible, are essential before their widespread use in everyday clinical practice.

Finally, malignant tumors exhibit major phenotypic differences that can be visualized noninvasively by medical imaging. Innovations in medical devices (hardware) and image analysis (software) are moving the field toward quantitative imaging and shall likely improve the ability to evaluate tumor heterogeneity, a characteristic that has been linked to more aggressive tumor behavior (*e.g.*, resistance to treatment and development of metastases) [104]. Radiomics, or the high-throughput extraction of large numbers of image-based features, is certainly an exciting field in development that may potentially be correlated to genomic and proteomic patterns [105, 106]. The ability of imaging to quantify the spatial variation in architecture and function of individual tumors will likely become an essential tool for physicians to make therapeutic decisions in the near future.
